# Editorial: Computational and Experimental Insights in Redox-Coupled Proton Pumping in Proteins

**DOI:** 10.3389/fchem.2021.762612

**Published:** 2021-09-15

**Authors:** Vivek Sharma, Petra Imhof, Petra Hellwig

**Affiliations:** ^1^ University of Helsinki, Helsinki, Finland; ^2^ Friedrich-Alexander-Universität (FAU) Erlangen-Nürnberg, Erlangen, Germany; ^3^ Université de Strasbourg, Strasbourg, France

**Keywords:** cell respiration, photosynthesis, proton-coupled electron donor, molecular dynamics simulation, quantum chemical calculation, spectroscopy, mitochondria, reactive oxygen species (ROS)

Elementary electron and proton transfer reactions commonly occur in chemistry and biology. Proteins involved in oxidative- and photo-phosphorylation carry out these reactions to generate energy in the form of ATP. Despite the simplicity of electron and proton transfer reactions, these pose extreme challenge to study either by experimental or computational approaches. In this special issue, we present a collection of reviews, original research articles as well as perspectives written by top-level experimental and computational experts working in the field of redox-active enzymes and associated fields. The special issue presents a current state-of-the-art in our understanding of the mechanism of bioenergetic enzymes, and at the same time provides important glimpses of theoretical and experimental methodological advances in the field.

The respiratory complex I, NADH:quinone (Q) oxidoreductase, is the first electron acceptor of the electron transport chain (ETC) in many organisms and pumps protons by conserving energy from the reduction of quinone to quinol (Parey et al., 2020). Despite recent major advances in structural characterization of this large complex (500–1 MDa), the molecular mechanism of redox-coupled proton pumping remains largely unknown and among other questions, the role of quinone/quinol binding in the electrostatic and conformational control of enzyme remains unclear (Hielscher et al., 2013; Haapanen and Sharma, 2021). One of the central elements of the coupling mechanism is the interface between the peripheral “arm” catalysing electron transfer and a membrane “arm” responsible for proton translocation. In this special issue, Yoga et al. and Nuber et al. have reviewed and discussed interesting aspects of quinone/quinol binding and its coupling to the conformational changes in this critical region of complex I. Yoga et al. reviewed the latest structural and computational data on the binding of quinone in the unique ∼30 Å long quinone-binding tunnel of complex I, and discussed recent mechanistic models of proton pumping. Nuber et al. have highlighted the importance of movement of quinol (QH^−^) anion in the quinone tunnel and protonation/deprotonation reactions in the redox-coupled proton pumping mechanism of complex I.

The third complex in the ETC is complex III and is described by a well-known Q cycle (Crofts et al., 2017). Husen and Solov’yov gave new insights into the side-reactions of complex III, and by performing multiscale computational simulations they suggest how superoxide forms by reaction between dioxygen and semiquinone, and how it is released to the membrane-solvent environment. Thus, shedding light on the ROS (reactive oxygen species) generation by complex III of the ETC. Sarewicz et al. by performing site-directed mutagenesis of heme *b*_L_ ligand and spectroscopic measurements provided new insights into the redox reactions of Q_o_ site of complex III.

The final electron acceptor, complex IV, acts as an electron sink in the ETC of many organisms, and efficiently pumps protons by conserving the free energy of oxygen reduction (Wikström and Sharma, 2018). The functional importance of a tyrosyl radical in the catalytic cycle of complex IV has been emphasized based on computations and experiments (Voicescu et al., 2009; Sharma et al., 2013). Blomberg, based on hybrid density functional theory calculations (Blomberg) discusses how highly conserved redox-active tyrosine remains deprotonated until the last steps of the catalytic cycle, a notion that is central to drive proton pumping even at high proton motive force. Baserga et al. performed advanced FTIR spectroelectrochemical titrations and provided a quantitative description of the changes in electric field at the active site of complex IV during its redox reactions.

Kaur and colleagues provide a holistic view on the proton binding motifs of the redox-active proton pumps. They highlight the central role played by *proton loading sites* (sites in protein that uptake and release protons) in maintaining pumping even at high proton motive force. Relatedly, Bondar presents a comparison of several proton translocating enzymes and their proton binding motifs, and emphasizes the importance of these in understanding mechanism of proton pumping enzymes. In the study of Calisto and Pereira, sequence and structural analysis of NrfD-like subunits is presented and their role in ion-translocation and quinone binding is discussed.

Other complex electron and proton translocating enzymes are presented, completing the picture on the complexity of coupled electron and proton translocation in biological systems. Wu et al. reveal the complex electron transfer in NADPH oxidase 5 (NOX5), a member of a family of enzymes, dedicated to the production of reactive oxygen species. By computer simulations, they analysed the O_2_ movement and electron tunneling pathways in the inter-heme electron-transfer steps that ultimately lead to production of superoxide in NOX5. Finally, Dale-Evans et al. describe the mechanism of HypD from *E. coli* by means of theoretical and computational studies on the voltametric current. Their data allowed them to specifically estimate the kinetic parameter and reveal a step-wise one-electron, one-proton transfer mechanism rather than a concerted two-electron redox reaction.

The work presented in this special issue highlights how experimental (spectroscopy, biochemistry, etc.) and computational (classical and quantum chemical simulations) studies in concert have greatly advanced our knowledge of proton-coupled electron transfer processes. Even with the broad range of exciting results and mechanistic understanding already obtained, the complexity of the systems involved in redox-coupled proton pumping is so high that novel developments at all levels will be needed in years and decades to come.
